# Taxonomy and phylogeny of *Resinicium sensu lato* from Asia-Pacific revealing a new genus and five new species (*Hymenochaetales*, *Basidiomycota*)

**DOI:** 10.1186/s43008-021-00071-1

**Published:** 2021-07-19

**Authors:** Jia Yu, Xue-Wei Wang, Shi-Liang Liu, Shan Shen, Li-Wei Zhou

**Affiliations:** 1 School of life Science, Liaoning University, Shenyang, 110036 Liaoning China; 2 State Key Laboratory of Mycology, Institute of Microbiology, Chinese Academy of Sciences, Beijing, 100101 China; 3 University of Chinese Academy of Sciences, Beijing, 100049 China

**Keywords:** Corticioid fungi, *Skvortzovia*, *Skvortzoviella*, Wood-inhabiting fungi, Six new taxa

## Abstract

*Resinicium*, belonging to *Hymenochaetales*, *Agaricomycetes*, is a worldwide genus of corticioid wood-inhabiting fungi. To improve the knowledge of species diversity within the *Hymenochaetales*, two dozen specimens from Asia-Pacific preliminarily identified to be members of *Resinicium sensu lato* were carefully studied from morphological and phylogenetic perspectives. From these specimens, a new monotypic genus *Skvortzoviella*, and five new species, viz. *Resinicium austroasianum*, *R. lateastrocystidium*, *Skvortzovia dabieshanensis*, *S. qilianensis* and *Skvortzoviella lenis* are described; moreover, a new basal lineage of *Resinicium* represented by a Vietnam specimen and three Chinese specimens of *S. pinicola* are identified. The six newly proposed taxa are morphologically compared with related genera and species, while the family positions of *Resinicium*, *Skvortzovia,* and *Skvortzoviella* within the *Hymenochaetales* are still ambiguous. In addition, the ancestral geographic origin of *Resinicium*, even though inconclusive, is now thought to be Asia-Pacific instead of tropical America as previously assumed.

## INTRODUCTION

*Resinicium*, a worldwide genus of corticioid wood-inhabiting fungi, was erected for *Hydnum bicolor* and *Corticium furfuraceum* with the former as the generic type (Parmasto 1968). Although this genus is treated as a member of *Rickenellaceae* within *Hymenochaetales* (He et al. 2019; Olariaga et al. 2020), the corresponding phylogenetic analysis did not have a comprehensive sampling throughout this order (Olariaga et al. 2020). Due to the ambiguous circumscription of families within *Hymenochaetales*, the taxonomic position of *Resinicium* at the family level was not fully clarified from the phylogenetic perspective.

A total of 22 species have been assigned to *Resinicium* at some stage (Index Fungorum: http://www.indexfungorum.org/Names/Names.asp). However, phylogenetic analyses have indicated that *Resinicium* is not a monophyletic genus (Larsson et al. 2006; Nakasone 2007). Within *Hymenochaetales*, *R. aculeatum*, *R. bicolor*, *R. confertum*, *R. friabile*, *R. grandisporum*, *R. monticola*, *R. mutabile*, *R. rimulosum*, *R. saccharicola*, and *R. tenue* were accepted to be members of *Resinicium sensu stricto*, whereas *R. furfuraceum*, *R. furfurellum*, *R. meridionale* and *R. pinicola* were included in the clade of *Resinicium sensu lato* and now are put in *Skvortzovia* (Larsson et al. 2006; Nakasone 2007; Telleria et al. 2008; Gruhn et al. 2017; Gruhn and Hallenberg 2018). *Skvortzovia* was originally erected as a monotypic genus for *Odontia furfurella* (Hjortstam and Bononi 1987). Besides the above-mentioned four species, *Phlebia georgica* was also combined into *Skvortzovia*, bringing the number of species in that genus to five (Gruhn and Hallenberg 2018).

Morphologically, *Resinicium* is characterized by resupinate, thin, soft basidiomes with smooth to odontioid hymenia, a monomitic hyphal system mainly with clamp connections or with simple septa in few species, the presence of astrocystidia, and thin-walled, smooth, ellipsoid to cylindrical basidiospores. *Skvortzovia* is quite similar to *Resinicium* but differs in the absence of astrocystidia.

In this study, we focus on *Resinicium sensu lato*. represented by specimens from the Asia-Pacific region including China, Vietnam, Malaysia, and Australia. Two new species are described in each of *Resinicium* and *Skvortzovia*, while a new monotypic genus typified by a new species without a confirmed position at the family level is introduced.

## MATERIALS AND METHODS

### Morphological examination

The studied specimens are deposited at the Fungarium, Institute of Microbiology, Chinese Academy of Sciences (HMAS), Beijing, China. The specimens were observed with Leica M125 (Wetzlar, Germany) and Nikon SMZ 1500 (Tokyo, Japan) stereomicroscopes and an Olympus BX 43 light microscope (Tokyo, Japan) at magnifications up to 1000×. Special color terms follow Petersen (1996). Microscopic procedures followed Wang et al. (2020). Specimen sections were mounted in Cotton Blue (CB), Melzer’s reagent (IKI), and 5% potassium hydroxide (KOH). All measurements were made from materials in CB. When presenting the variation of basidiospore sizes, 5% of the measurements were excluded from each end of the range and are given in parentheses. Drawings were made with the aid of a drawing tube. The following abbreviations are used in the descriptions: L = mean basidiospore length (arithmetic average of all measured basidiospores), W = mean basidiospore width (arithmetic average of all measured basidiospores), Q = variation in the L/W ratios between the studied specimens, and (a/b) = number of basidiospores (a) measured from given number (b) of specimens.

### Molecular sequencing

Crude DNA was extracted from dry specimens as templates for subsequent PCR amplification using CTAB rapid plant genome extraction kit-DN14 (Aidlab Biotechnologies, Beijing, China). The primer pairs ITS5/ITS4 (White et al. 1990; Gardes and Bruns 1993) and LR0R/LR7 (Vilgalys and Hester 1990) were selected for amplifying ITS and nLSU regions, respectively. The PCR procedures were as follows: for ITS region, initial denaturation at 95 °C for 3 min, followed by 34 cycles at 94 °C for 40 s, 57.2 °C for 45 s and 72 °C for 1 min, and a final extension at 72 °C for 10 min; for nLSU region, initial denaturation at 94 °C for 1 min, followed by 34 cycles at 94 °C for 30 s, 47.2 °C for 1 min and 72 °C for 1.5 min, and a final extension at 72 °C for 10 min. The PCR products were sequenced with the same primers used in PCR amplification at the Beijing Genomics Institute, Beijing, China. All newly generated sequences are deposited in GenBank (https://www.ncbi.nlm.nih.gov/genbank/; Table 1).

**Table 1:** Information of species used in phylogenetic analyses

Order	Species	Voucher	GenBank accession number	
			ITS	LSU
*Auriculariales*	*Auricularia brasiliana*	URM 85567	NR151845	NG057066
*Hymenochaetales*	*Alloclavaria purpurea*	T.Niskanen 01-053	MF319053	MF318894
	*Atheloderma mirabile*	TAA 169235	DQ873592	DQ873592
	*Basidioradulum radula*	AFTOL-ID 451	DQ234537	AY700184
	*Blasiphalia pseudogrisella*	P.Hoijer 4539	MF319045	MF318896
	*Bridgeoporus sinensis*	Cui 10,013	KY131832	KY131891
	*Coltricia perennis*	Cui 10,319	KU360687	KU360653
	*Contumyces rosellus*	Redhead 7501	U66452	U66452
	*Fasciodontia brasiliensis*	MSK-F 7245a	MK575201	MK598734
	*Fibricium rude*	CBS 339.66	MH858815	MH870454
	*Fomitiporia hartigii*	Cui 9914	KY750527	MT319381
	*Globulicium hiemale*	Hjm 19,007	DQ873595	DQ873595
	*Gyroflexus brevibasidiatus*	Lutzoni 930,826-1	U66441	U66441
	*Hastodontia halonata*	HHB-17058	MK575207	MK598738
	*Hymenochaete rubiginosa*	He 1049	JQ716407	JQ279667
	*Hyphodontia alutaria*	KHL 11889	DQ873603	DQ873603
	*Kneiffiella abieticola*	KHL 12498	DQ873601	DQ873601
	*Leucophellinus hobsonii*	Cui 6468	KT203288	KT203309
	*Loreleia marchantiae*	Lutzoni 910826-1	U66432	U66432
	*Lyomyces griseliniaea*	KHL 12971	DQ873651	DQ873651
	*Muscinupta laevis*	JJ 020909	EU118621	EU118621
	*Neoantrodiella gypsea*	Cui 10372	KT203290	MT319396
	*Nigrofomes melanoporus*	Vlasak 1704-39	MF629835	MF629831
	*Odonticium romellii*	KHL 1514b	DQ873639	DQ873639
	*Peniophorella praetermissa*	KHL 13164	DQ873597	DQ873597
	*Phellinidium ferrugineofuscum*	Cui 10042	KR350573	MT319388
	*Repetobasidium conicum*	KHL 12338	DQ873647	DQ873647
	***Resinicium austroasianum***	**LWZ 20171014-3**	**MW414503**	**MW414449**
		**LWZ 20180417-5**	**MW414504**	**MW414450**
		**LWZ 20180417-28**	**MW414505**	**MW414451**
		**LWZ 20180517-42**	**MW414506**	**MW414452**
		**LWZ 20180518-2**	**MW414507**	**MW414453**
	*R. bicolor*	O.Miettinen 14049	MF319079	MF319009
		FP-133575	DQ826533	
		HHB10731	DQ826534	
		JLL13731	DQ826535	
		FP-133695	DQ826536	
		HHB 10108	DQ826537	
		AFTOL-ID 810	DQ218310	
		O3	JQ765682	
		UC2022858	KP814209	
		TENN57741	AF518763	
		GEL2071	DQ340321	
		Z-3-4	FJ872065	
	*R. confertum*	FP-102863	DQ826538	
	*R. friabile*	FP-102983	DQ826545	DQ863690
		CBS 126043	MH864058	MH875513
		FP-102803	DQ826541	
		PR-1380	DQ826542	
		FP-150153	DQ826543	
		ECCO-146	DQ826544	
		MS77	KJ831948	
	*R. grandisporum*	GGGUY13-008	KY995325	
		GGGUY13-030	KY995326	
		GGGUY13-031	KY995327	
		GGMAR12-326	KY995329	
	***R. lateastrocystidium***	**LWZ 20180414-13**	**MW414508**	**MW414454**
		**LWZ 20180414-15**	**MW414509**	**MW414455**
		**LWZ 20180416-10**	**MW414510**	**MW414456**
	*R. monticola*	FP-150360	DQ826552	DQ863697
		FP-102832	DQ826550	
		FP-150061	DQ826551	
		FP-150355	DQ826553	
		FP-150407	DQ826554	
		FP-150332	DQ826555	
	*R. mutabile*	FP-102989	DQ826556	DQ863699
		PR-1366	DQ826557	
		GGGUY12-087	KY995322	
		GGMAR15-174	KY995330	
		GGMAR15-175	KY995331	
	*R. rimulosum*	FP-150328	DQ826546	
		KUC20131022-12	KJ668464	
	*R. saccharicola*	FP-102754	DQ826547	DQ863691
		FP-102841	DQ826548	
		FP-102843	DQ826549	
		GGGUY12-118	KY995323	
		GGGUY12-158	KY995324	
		GGMAR12-230	KY995328	
	*R. tenue*	FP-150354	DQ826539	
		FP-150251	DQ826540	
	*R.* sp.	026	KC785591	
	*R.* sp.	ASR-272	GU973812	
	*R.* sp.	ASR-290	GU973828	
	*R.* sp.	GSR1	FJ179463	
	***R.*** **sp.**	**LWZ 20171015**-**31**	**MW414511**	**MW414457**
	*Rickenella fibula*	P.Salo 1882	MF319088	MF319027
	*Rigidoporus corticola*	Dai 12632	KF111018	KF111020
	*Sidera lunata*	JS 15063	DQ873593	DQ873593
	***Skvortzovia dabieshanensis***	**LWZ 20201012**-**22**	**MW414512**	**MW414458**
		**LWZ 20201014**-**18**	**MW414513**	**MW414459**
		**LWZ 20201017**-**55**	**MW414514**	**MW414460**
	*S. furfuracea*	KHL 11738	DQ873648	DQ873648
	*S. furfurella*	KHL 10180	DQ873649	DQ873649
	*S. georgica*	KHL 12019	DQ873645	DQ873645
	*S. pinicola*	KHL 12224	DQ873637	DQ873637
		**LWZ 20180921**-**6**	**MW414515**	**MW414461**
		**LWZ 20201011**-**18**	**MW414516**	**MW414462**
		**LWZ 20201013**-**15**	**MW414517**	**MW414463**
	***S. qilianensis***	**LWZ 20180904**-**16**	**MW414518**	**MW414464**
		**LWZ 20180904**-**18**	**MW414519**	**MW414465**
		**LWZ 20180904**-**20**	**MW414520**	**MW414466**
	***Skvortzoviella lenis***	**LWZ 20180921**-**7**	**MW414521**	**MW414467**
		**LWZ 20180921**-**17**	**MW414522**	**MW414468**
		**LWZ 20180921**-**25**	**MW414523**	**MW414469**
		**LWZ 20180921**-**32**	**MW414524**	**MW414470**
		**LWZ 20180922**-**39**	**MW414525**	**MW414471**
		**LWZ 20180922**-**61**	**MW414526**	**MW414472**
	*Sphaerobasidium minutum*	KHL 11714	DQ873652	DQ873653
	*Trichaptum abietinum*	NH 12842	AF347104	AF347104
	*Tubulicrinis globisporus*	KHL 12133	DQ873655	DQ873655
	*T. hirtellus*	KHL 11717	DQ873657	DQ873657
	*Xylodon asperus*	KG Nilsson 2004b	DQ873606	DQ873607
*Polyporales*	*Abortiporus biennis*	TFRI 274	EU232187	EU232277
	*Antrodiella semisupina*	FCUG 960	EU232182	EU232266
	*Earliella scabrosa*	PR1209	JN165009	JN164793
	*Fomitopsis betulina*	Otto Miettinen 12388	JQ700297	JQ700297
	*Fragiliporia fragilis*	Dai 13080	KJ734260	KJ734264
		Dai 13559	KJ734261	KJ734265
	*Melanoporia nigra*	X1735	KC543172	KC543172
	*Polyporus tuberaster*	Wei 2577	AB474086	KX900131
	*Radulodon aneirinus*	MUAF 888	EU340895	EU368503

The newly sequenced vouchers are in bold

### Phylogenetic analyses

Besides the newly generated sequences, additional related sequences were also downloaded from GenBank (Table 1) for inclusion in the phylogenetic analyses. Firstly, the combined dataset of ITS and nLSU regions (1) was used to explore the phylogenetic positions of the newly studied specimens within *Hymenochaetales*. All vouchers of *Hymenochaetales* and *Polyporales* listed in Table 1, each with both ITS and nLSU sequences available, were included as ingroup taxa, while *Auricularia cornea* from *Auriculariales* was selected as an outgroup taxon (Hibbett et al. 2007). Due to previous phylogenetic studies focusing on *Resinicium* being mainly based solely on the ITS region, a voucher- and species-abundant ITS dataset of *Resinicium* (2), comprising all vouchers of *Resinicium* in Table 1, was used to further differentiate species identities within this genus*.* Finally, another combined dataset of ITS and nLSU regions (3) was used to perform a biogeographic analysis of *Resinicium*. All vouchers of *Resinicium* listed in Table 1, each with both ITS and nLSU sequences available, were included in this dataset. No outgroup taxa were selected for datasets 2 and 3.

All datasets were aligned using MAFFT 7.110 (Katoh and Standley 2013) under the G-INS-i option (Katoh et al. 2005). Regarding the combined datasets of the ITS and nLSU regions, each region was aligned separately and then the alignments of the two regions were concatenated as a single alignment. The best-fit evolutionary models of alignments for phylogenetic analyses were estimated using jModelTest (Guindon and Gascuel 2003; Posada 2008) under Akaike information criterion.

Regarding datasets 1 and 2, Maximum Likelihood (ML) and Bayesian Inference (BI) methods were utilized for phylogenetic analyses. The ML method was conducted using raxmlGUI 1.2 (Silvestro and Michalak 2012; Stamatakis 2006) with calculation of bootstrap (BS) replicates under the auto FC option (Pattengale et al. 2010). The BI method was conducted using MrBayes 3.2 (Ronquist et al. 2012). Two independent runs were employed, and each run had four chains and started from random trees. Trees were sampled every 1000th generation, and the first 25% of trees were removed, while the other 75% of trees were retained for constructing a 50% majority consensus tree and calculating Bayesian posterior probabilities (BPPs). Tracer 1.5 (http://tree.bio.ed.ac.uk/software/tracer/) was used to judge whether chains converged.

A consensus tree for the alignment resulting from dataset 3 was generated by BI method using BEAST v1.10.4 (Suchard et al. 2018). Trees were sampled every 1000th generation from a total of 50 million generations and the first 10% of the sampled trees were removed as burn-in. Chain convergence recorded in the resulting log file was determined using Tracer 1.5. The consensus tree was used for biogeographic analysis using RASP 4.2 under the Bayesian Binary MCMC (BBM) analysis with default parameters (Yu et al. 2015, 2020). Six geographic origins, viz. Asia-Pacific, Europe, North America, South America, tropical America and Africa were set according to voucher information.

## RESULTS

A total of 24 specimens preliminarily identified to *Resinicium sensu lato* were studied further. ITS and nLSU regions were newly generated from all these specimens (Table 1).

**Fig. 1:**
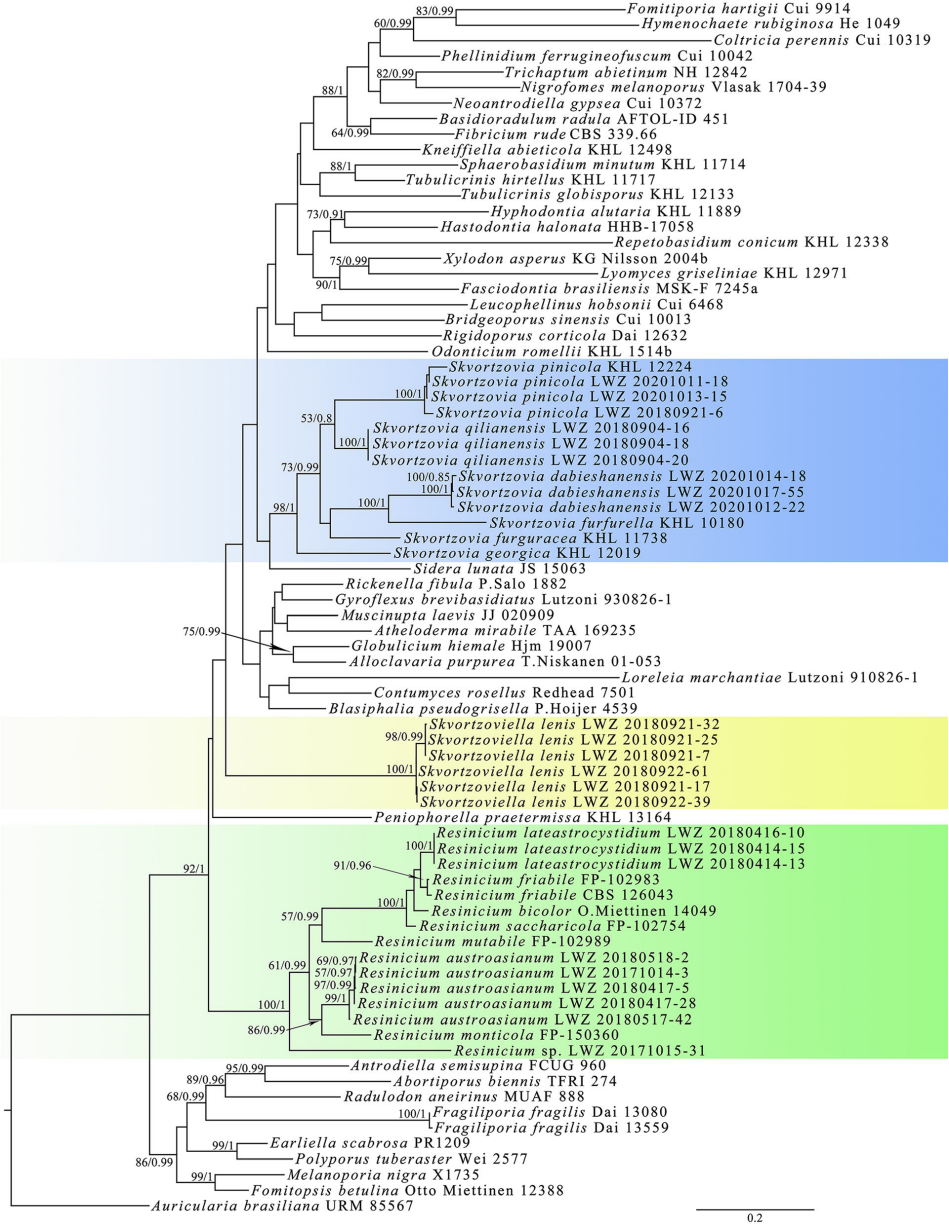
Phylogenetic positions of the newly studied specimens of *Resinicium sensu lato* within the *Hymenochaetales* inferred from the combined dataset of ITS and nLSU regions. The topology generated by the maximum likelihood method is presented along with the bootstrap values and the Bayesian posterior probabilities above 50% and 0.8, respectively, at the nodes

The combined dataset of ITS and nLSU regions (1) from 78 collections generated a concatenated alignment of 2399 characters with GTR + I + G as the best-fit evolutionary model. The ML search stopped after 250 BS replicates. In BI, all chains converged after 50 million generations with an average standard deviation of split frequencies of 0.002644, which was indicated by all effective sample sizes (ESSs) above 13,600 and the potential scale reduction factors (PSRFs) close to 1.000. ML and BI methods generated similar topologies in main lineages, and thus only the topology generated by the ML method is presented along with BS values and BPPs above 50% and 0.8, respectively, at the nodes (Fig. 1). The phylogeny generated by this dataset well supported *Hymenochaetales* as an independent order (BS = 92%, BPP = 1). Within *Hymenochaetales*, the family rank was not resolved, whereas at the generic rank *Resinicium* was fully supported and *Skvortzovia* was strongly supported (BS = 98%, BPP = 1). In the genera *Resinicium* and *Skvortzovia*, three (one including a single specimen LWZ 20171015-31 from Vietnam) and two new lineages, respectively, emerged, and three studied specimens were grouped with *Skvortzovia pinicola* with full support. Moreover, an independent clade from other sampled genera and species composed of six newly studied specimens was also fully supported.

**Fig. 2:**
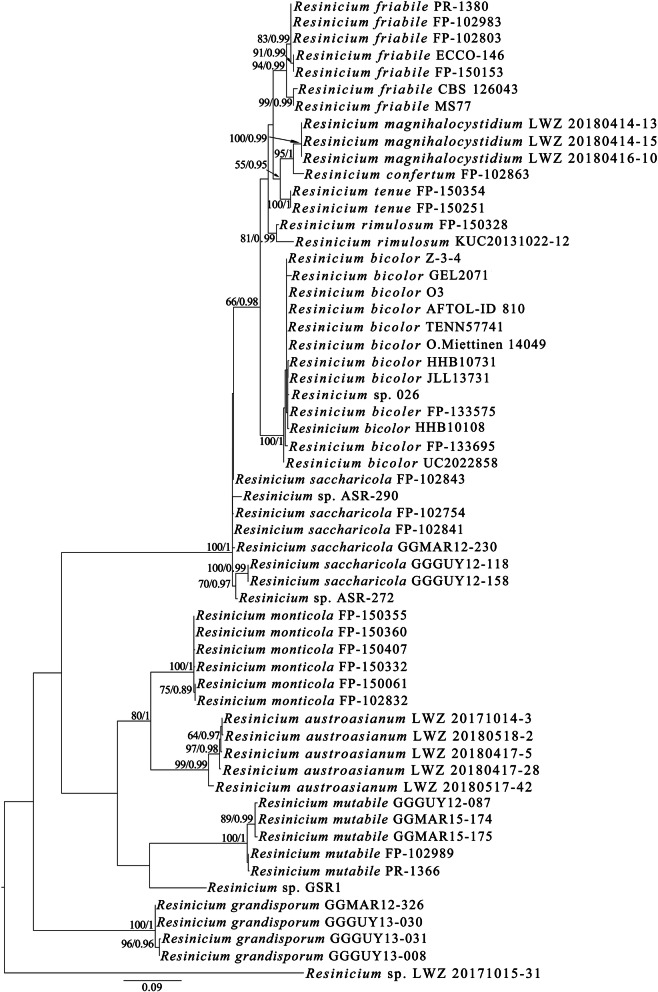
Species identities of *Resinicium* differentiated by ITS-based phylogeny. The midpoint-rooted tree generated by the maximum likelihood method is presented along with the bootstrap values and the Bayesian posterior probabilities above 50% and 0.8, respectively, at the nodes

The ITS dataset of *Resinicium* (2) from 58 collections generated an alignment of 645 characters with GTR + I + G as the best-fit evolutionary model. The ML search stopped after 300 BS replicates. In BI, after 10 million generations, all chains converged with an average standard deviation of split frequencies of 0.002680, which was indicated by all ESSs above 5500 and the PSRFs equal to 1.000. ML and BI methods generated similar topologies in main lineages. Therefore, the topology inferred from ML method was presented along with BS values and BPPs above 50% and 0.8, respectively, at the nodes (Fig. 2). The midpoint-rooted tree recovered nine known species of *Resinicium*, while the newly studied specimens formed three independent lineages (one including a single specimen LWZ 20171015-31 from Vietnam) as in the phylogeny inferred from dataset 1.

Taking both morphological characters and the phylogenies inferred from datasets 1 and 2 into consideration, two new species from each of *Resinicium* and *Skvortzovia*, and a new monotypic genus typified by a new species within *Hymenochaetales* are described below. The new lineage with a single specimen LWZ 20171015-31 from Vietnam in *Resinicium* is treated as *R.* sp. instead of being described as a new species until more collections that group with LWZ 20171015-31 are available and carefully studied.

**Fig. 3:**
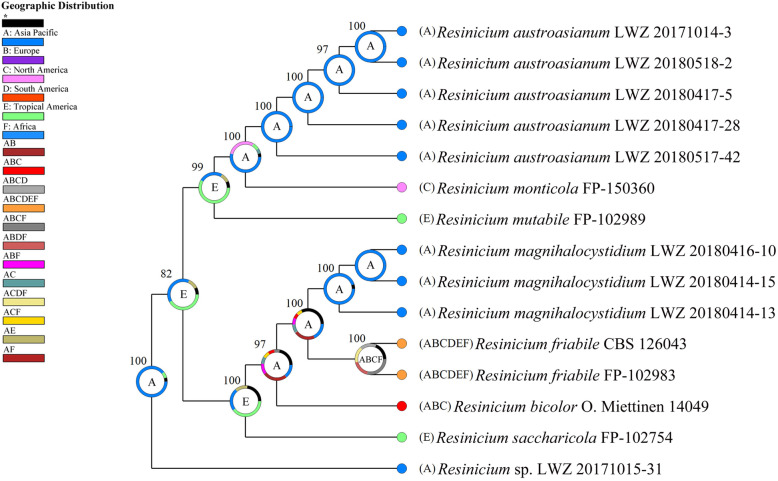
Biogeography of *Resinicium* inferred from the combined dataset of ITS and nLSU regions. The midpoint-rooted consensus tree was generated by the Bayesian inference method using BEAST. As evaluated using RASP under the Bayesian Binary MCMC analysis, the geographic origin was indicated by a pie chart at each node of the tree. The geographic distribution represented by each color and letter in the pie charts and the illustration is indicated in the upper left of the tree

Dataset 3 from 15 collections generated a concatenated alignment of 1457 characters. The best-fit evolutionary model for this alignment was estimated as GTR + I + G. Chain convergence was indicated by all ESSs above 3500. The midpoint-rooted phylogeny successfully resolved the species relationships within *Resinicium* and the ancestral geographic origin of *Resinicium* was estimated to be Asia-Pacific (Fig. 3).

## TAXONOMY

**Fig. 4:**
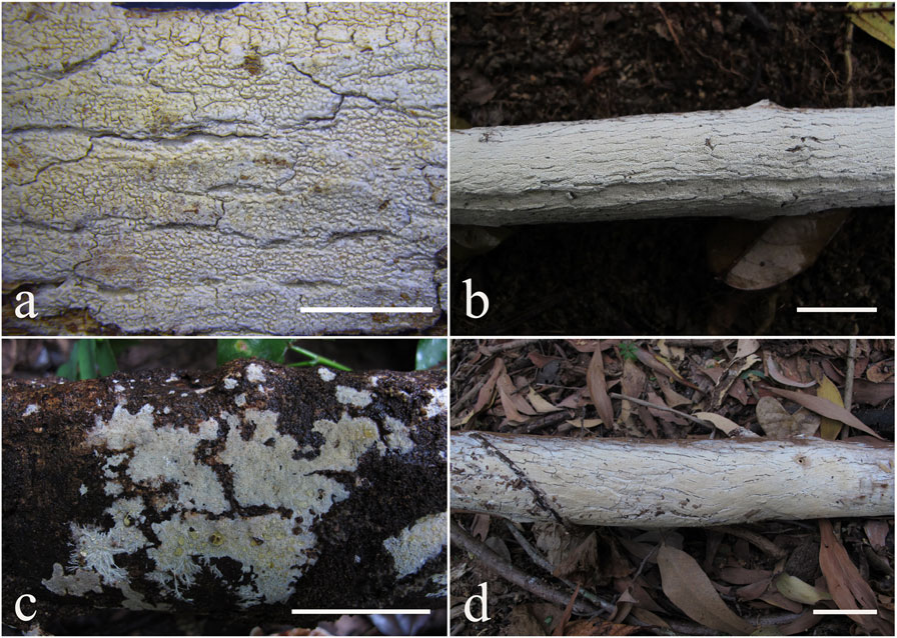
Basidiomes of *Resinicium austroasianum*. **a**, **b** LWZ 20180417-5 (holotype). **c** LWZ 20171014-3 (paratype). **d** LWZ 20180518-2 (paratype). Scale bars: **a** = 5 mm, **b**–**d** = 2 cm

**Fig. 5:**
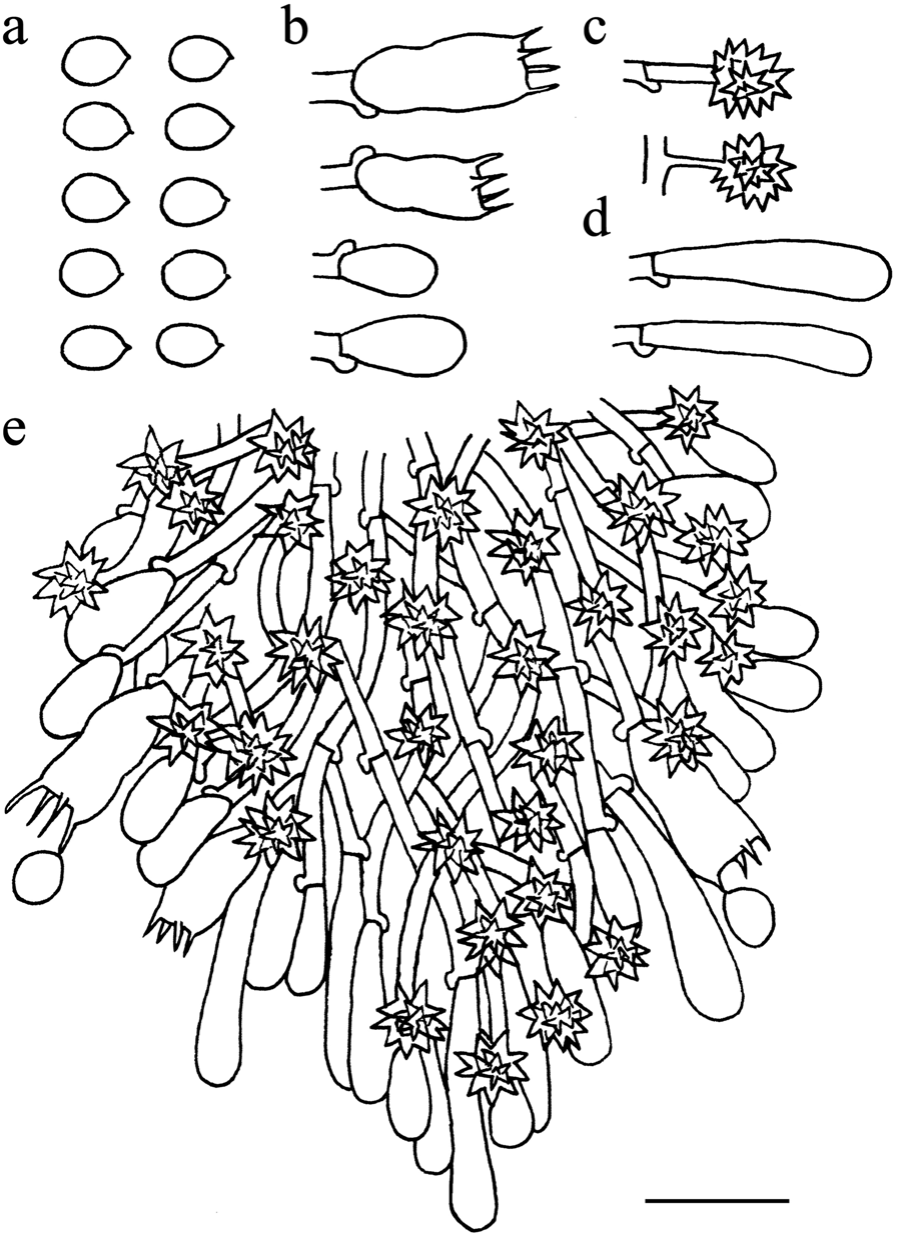
Microscopic structures of *Resinicium austroasianum* (drawn from holotype). **a** basidiospores. **b** basidia and basidioles. **c** astrocystidia. **d** leptocystidia. **e** apical part of a tooth. Scale bar = 10 μm

***Resinicium austroasianum*** Jia Yu, Xue W. Wang, S.L. Liu & L.W. Zhou, **sp. nov**. (Figs. 4–5)

MycoBank: MB 840227.

*Etymology: austroasianum* (Lat.), refers to South Asia.

*Diagnosis:* Similar to *R. monticola* in the absence of halocystidia; however, *R. monticola* differs by its narrower basidiospores (2.8–3.2 μm in width) and rarely cracked basidiomes (Nakasone 2007).

*Type:* **Malaysia**: *Selangor*: Kota Damansara Community Forest Reserve, on fallen angiosperm branch, 17 Apr. 2018, *Li-Wei Zhou*, *LWZ 20180417-5* (HMAS– holotype).

*Description: Basidiomes* annual, resupinate, closely adnate, widely effused, not easily separable, thin, crustose, white to cream when fresh, pale yellow to curry-yellow with age, usually cracked into polygons. *Hymenophore* grandinioid to odontoid, composed of small cylindrical aculei, usually with round apex, 5–6 per mm, 300–500 μm long. *Subiculum* not stratified, white, 50–100 μm thick. Margin gradually thinning out, white, occasionally with mycelial cords.

*Hyphal system* monomitic, generative hyphae with clamp connections. *Subiculum* composed of crystal clusters and agglutinated hyphae; subicular hyphae hyaline, thin-walled, frequently branched, often collapsed and indistinct, sometimes with denuded spines, 2–3 μm diam. Subhymenial hyphae obscured from numerous crystal clusters, frequently branched, hyaline, compact and agglutinated, 1.5–2.5 μm diam. *Astrocystidia* extremely abundant in hymenium and subhymenium, often developing both terminally and laterally on hyphae, hyaline, thin-walled, 7–20 × 1–3 μm, 1–1.5 μm diam at base, at apex a stellate cluster of hyaline crystals, up to 4–6 μm diam. *Hymenial leptocystidia* numerous, cylindrical with obtuse apex, 10–30 × 2–3 μm, with a basal clamp. *Basidia* cylindrical, often with a median constriction, four sterigmata, 10–20 × 4–6 μm, tapering to 2–3 um diam with a clamp connection at base; basidioles similar in shape to basidia, but smaller. *Basidiospores* ellipsoid, hyaline, smooth, thin-walled, acyanophilous, non-amyloid, non-dextrinoid, (4.2–)4.5–5.1(− 5.3) × (3.2–)3.3–4.2(− 4.3) μm, L = 4.8 μm, W = 3.7 μm, Q = 1.25–1.28 (*n* = 90/3).

*Additional specimens examined:* **Australia:** *Queensland*: Cairns: Cairns Botanic Gardens, on angiosperm stump, 17 May 2018, *Li-Wei Zhou*, *LWZ 20180517-42* (HMAS); Mount Whitfield Conservation Park, on fallen angiosperm branch, 18 May 2018, *Li-Wei Zhou*, *LWZ 20180518-2* (HMAS). – **Malaysia:** *Selangor*: Kota Damansara Community Forest Reserve, on angiosperm stump, 17 Apr. 2018, *Li-Wei Zhou*, *LWZ 20180417-28* (HMAS). – **Vietnam:** Thac Mai Preservation Park, on fallen angiosperm branch, 14 Oct. 2017, *Li-Wei Zhou*, *LWZ 20171014-3* (HMAS).

**Fig. 6:**
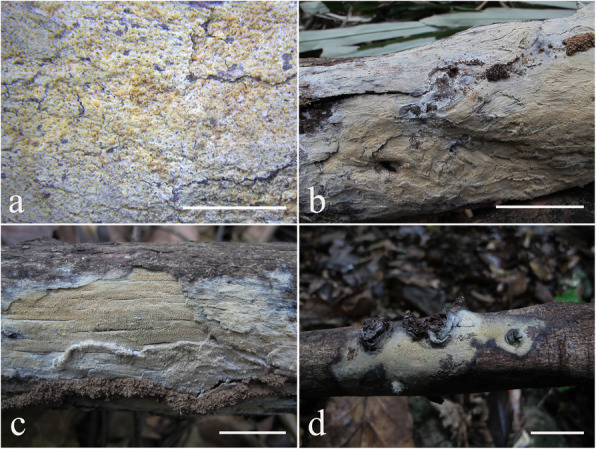
Basidiomes of *Resinicium lateastrocystidium*. **a**, **b** LWZ 20180414-15 (holotype). **c** LWZ 20180414-13 (paratype). **d** LWZ 20180416-10 (paratype). Scale bars: **a** = 5 mm, **b**–**d** = 2 cm

**Fig. 7:**
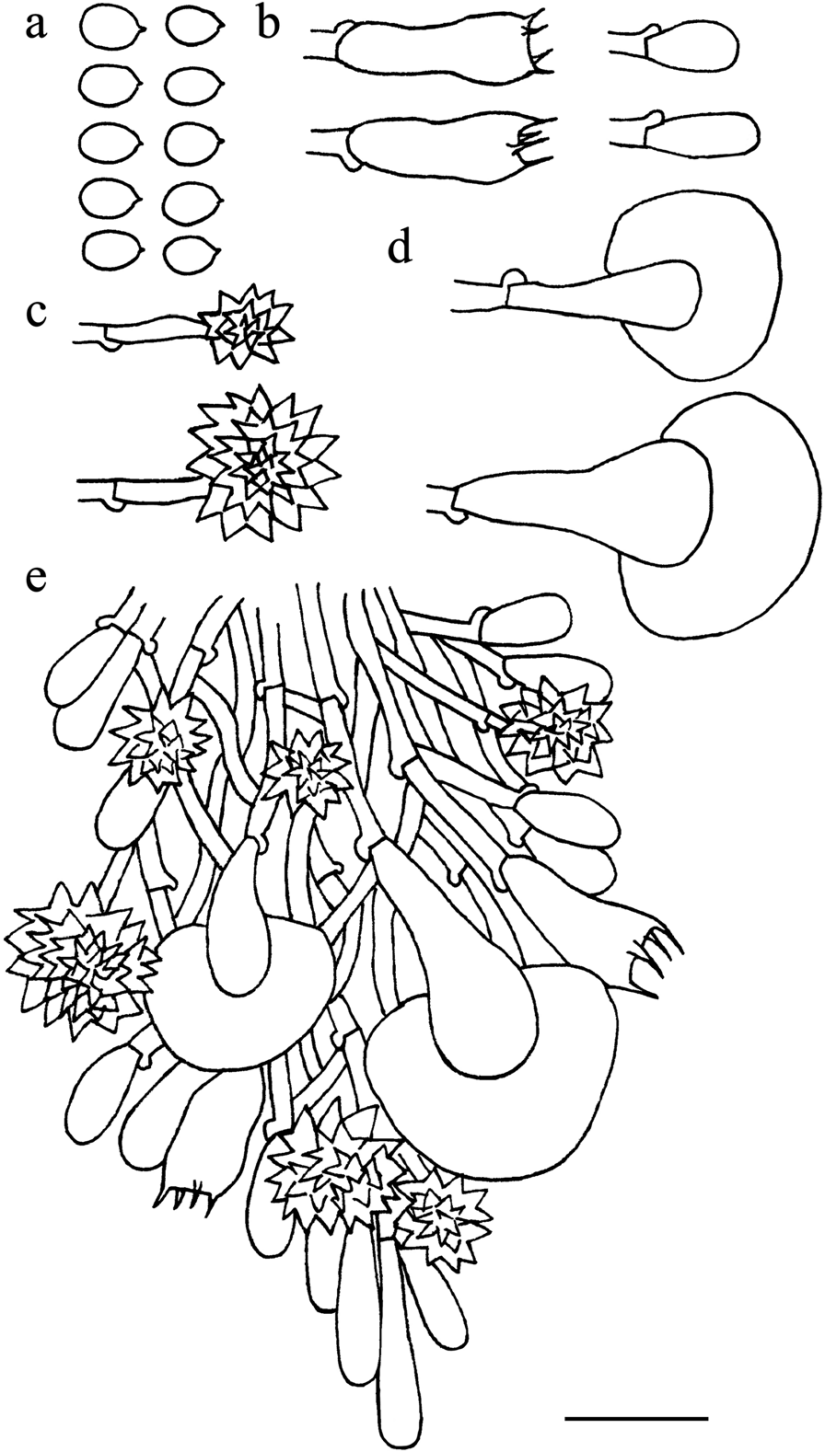
Microscopic structures of *Resinicium lateastrocystidium* (drawn from holotype). **a** basidiospores. **b** basidia and basidioles. **c** astrocystidia. **d** halocystidia. **e** apical part of a tooth. Scale bar = 10 μm

***Resinicium lateastrocystidium*** Jia Yu, Xue W. Wang, S.L. Liu & L.W. Zhou, **sp. nov.** (Figs. 6–7)

MycoBank: MB 840229.

*Etymology: lateastrocystidium* (Lat.), refers to the wide astrocystidia.

*Diagnosis:* Characterized in the genus by the presence of halocystidia and wide astrocystidia (above 6 μm in diam).

*Type:* **Malaysia:** *Kuala Lumpur*: KL Forest Eco park, on fallen angiosperm trunk, 14 Apr. 2018, *Li-Wei Zhou*, *LWZ 20180414-15* (HMAS – holotype).

*Description: Basidiomes* annual, resupinate, closely adnate, widely effused, not easily separable, thin, farinaceous, cream to buff-yellow when fresh, straw-yellow to olivaceous buff with age, not cracked. *Hymenophore* grandinioid to odontoid, usually with small conical apex, 3–4 per mm, 300–500 μm long. *Subiculum* not stratified, straw-yellow to olivaceous buff, 100–200 μm thick. *Margin* gradually thinning out, white, occasionally with mycelia cords.

*Hyphal system* monomitic, generative hyphae with clamp connections. Subiculum composed of mostly indistinct hyphae; subicular hyphae hyaline, thin-walled, moderately branched, often collapsed, 2–3 μm diam. *Subhymenial hyphae* frequently branched, hyaline, compact and agglutinated, 1.5–2.5 μm diam. *Astrocystidia* rare, scattered, often developing both terminally and laterally on hyphae, hyaline, thin-walled, 10–20 × 1.7–3 μm, 1.5–3 μm diam at base, at apex a stellate cluster of hyaline crystals, to 6–15 μm diam. *Halocystidia* hyaline, thin-walled, 15–30 × 4–10 μm, tapering to 2–3 μm diam at base, the outer layer inflates to a bladder, 10–20 μm diam, formed a capitate cystidium. *Basidia* cylindrical, often with a median constriction, four sterigmata, 13–20 × 5–6 μm, tapering to 2–4 um diam with a clamp connection at base; basidioles similar in shape to basidia, but smaller. *Basidiospores* ellipsoid, hyaline, smooth, thin-walled, acyanophilous, non-amyloid, non-dextrinoid, (3.9–)4.1–5 × (2.8–)2.9–3.9(− 4) μm, L = 4.5 μm, W = 3.3 μm, Q = 1.35–1.36 (*n* = 90/3).

*Additional specimens examined:* **Malaysia:** *Kuala Lumpur*: KL Forest Eco park, on fallen angiosperm trunk, 14 Apr. 2018, *Li-Wei Zhou*, *LWZ 20180414-13* (HMAS); *Selangor*: Kota Damansara Community Forest Reserve, on fallen angiosperm branch, 16 Apr. 2018, *Li-Wei Zhou*, *LWZ 20180416-10* (HMAS).

*Notes: Resinicium lateastrocystidium* resembles *R. friabile*, *R. rimulosum* and *R. saccharicola* in the presence of halocystidia and astrocystidia; however, astrocystidia in the latter three species are much narrower (1.5–3 μm in width in *R. friabile*, Nakasone 2007; 3–4 μm in width in *R. rimulosum*, Nakasone 2007; 1.5–2 μm in width in *R. saccharicola*, Nakasone 2000). In addition, these three species differ from *R. lateastrocystidium* by narrower or longer basidiospores (4.5–5 × 2.8–3 μm in *R. friabile*, Hjortstam and Melo 1997; 4–4.8 × 2.8–3 μm in *R. rimulosum*, Nakasone 2007; 5–6.1 × 3.3–4.2 μm in *R. saccharicola*, Nakasone 2000).

**Fig. 8:**
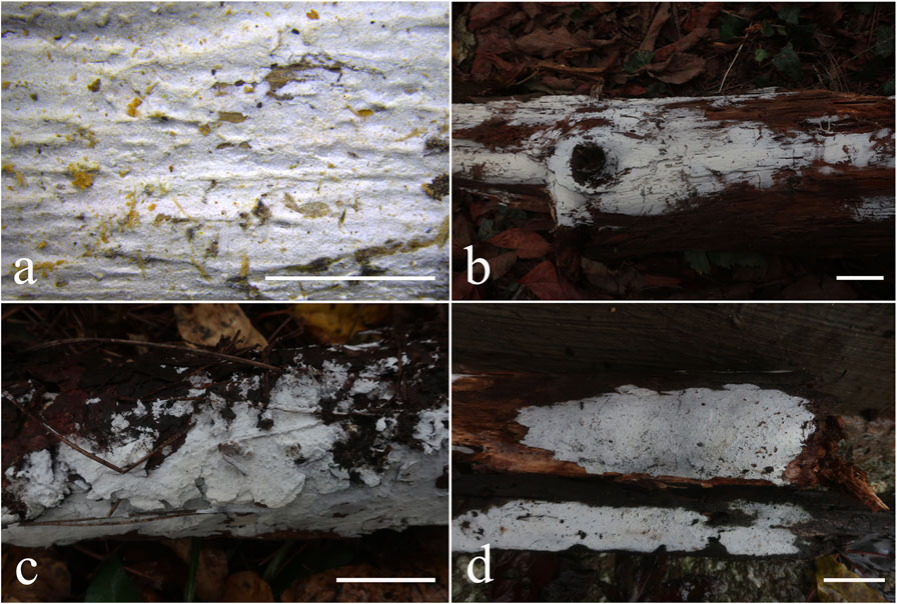
Basidiomes of *Skvortzovia dabieshanensis*. **a**, **b** LWZ 20201012-22 (holotype). **c** LWZ 20201014-18 (paratype). **d** LWZ 20201017-55 (paratype). Scale bars: **a** = 5 mm, **b**–**d** = 2 cm

**Fig. 9:**
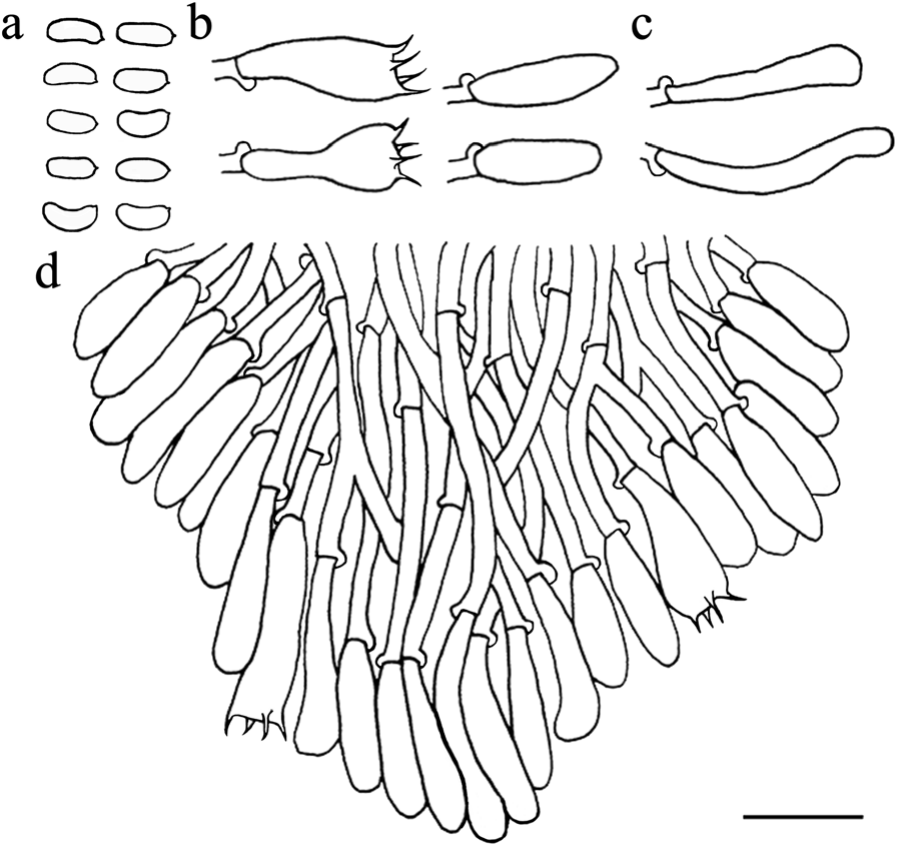
Microscopic structures of *Skvortzovia dabieshanensis* (drawn from holotype). **a** basidiospores. **b** basidia and basidioles. **c** leptocystidia. **d** apical part of a tooth. Scale bar = 10 μm

***Skvortzovia dabieshanensis*** Jia Yu, Xue W. Wang, S.L. Liu & L.W. Zhou, **sp. nov.** (Figs. 8–9)

MycoBank: MB 840228.

*Etymology: dabieshanensis* (Lat.), refers to the Dabieshan Mountains.

*Diagnosis*: Similar to *Skvortzovia furfurella* in the grey grandinioid basidiomes, capitate leptocystidia and lack of halocystidia, but *S. furfurella* differs by smaller allantoid basidiospores (3.5–4 × 0.8–1 μm, Gruhn and Hallenberg 2018).

*Type:* **China:** *Anhui*: Jinzhai County: Dabieshan Mountains: Tiantangzhai National Nature Reserve, on fallen trunk of *Pinus*, 12 Oct. 2020, *Li-Wei Zhou*, *LWZ 20201012-22* (HMAS – holotype).

*Description: Basidiomes* annual, resupinate, closely adnate, widely effused, not easily separable, thin, farinaceous, white to cream when fresh, ash-grey with age, not cracked. *Hymenophore* grandinioid, composed of small aculei, usually with round apex, 5–6 per mm, 200–300 μm long. *Subiculum* not stratified, white, 100–200 μm thick. *Margin* gradually thinning out, white, occasionally with mycelia cords.

*Hyphal system* monomitic, generative hyphae with clamp connections. Subiculum composed of indistinct generative hyphae; subicular hyphae hyaline, thin-walled, frequently branched, interwoven, 2–3 μm diam. *Subhymenial hyphae* hyaline, thin-walled, frequently branched, compact and agglutinated, 1.5–2.5 μm diam. *Hymenial leptocystidia* capitate, hyaline, thin-walled, with a basal clamp connection, 15–30 × 2.5–4 μm. *Basidia* clavate four sterigmata, 10–20 × 3.5–5 μm, tapering to 2–4 um diam with a clamp connection at base; basidioles similar in shape to basidia, but smaller. *Basidiospores* cylindrical to allantoid, hyaline, thin-walled, smooth, acyanophilous, non-amyloid, non-dextrinoid, (3.5–)3.8–4.7(− 4.9) × 1.8–2.4(− 2.6) μm, L = 4.2 μm, W = 2.1 μm, Q = 2.05–2.08 (*n* = 90/3).

*Additional specimen examined:* **China:** *Anhui*: Yuexi County: Dabieshan Mountains: Yaoluoping National Nature Reserve, on fallen trunk of *Pinus*, 14 Oct. 2020, *Li-Wei Zhou*, *LWZ 20201014-18* (HMAS); Shucheng County: Dabieshan Mountains: Wanfoshan National Nature Reserve, on fallen angiosperm trunk, 17 Oct. 2020, *Li-Wei Zhou*, *LWZ 20201017-55* (HMAS).

*Notes: Skvortzovia pinicola* has similar basidiospores in shape and size to *S. dabieshanensis* but differs by the presence of halocystidia in the apex of the aculei (Eriksson et al. 1981).

**Fig. 10:**
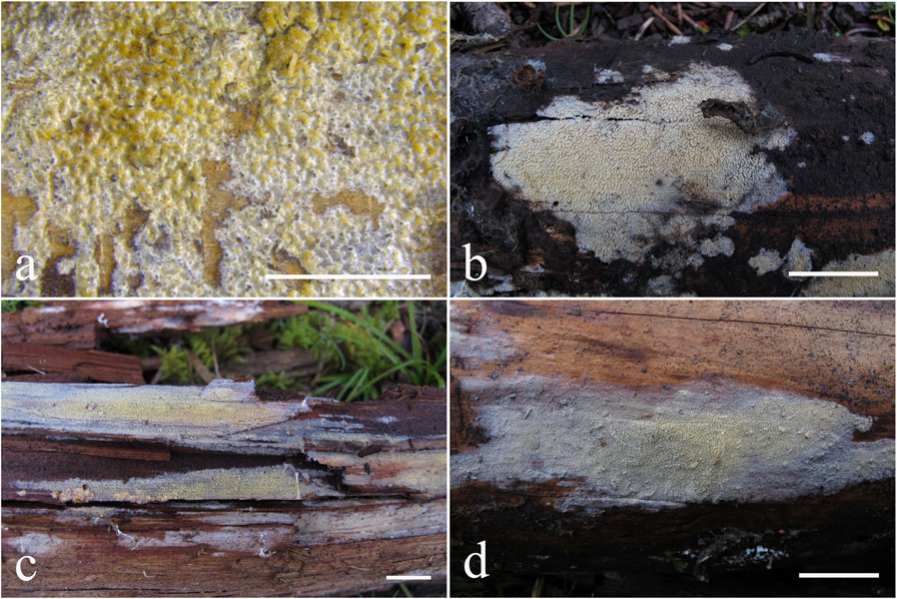
Basidiomes of *Skvortzovia qilianensis*. **a b** LWZ 20180904-16 (holotype). **c** LWZ 20180904-18 (paratype). **d** LWZ 20180904-20 (paratype). Scale bars: a = 5 mm, b–d = 2 cm

**Fig. 11:**
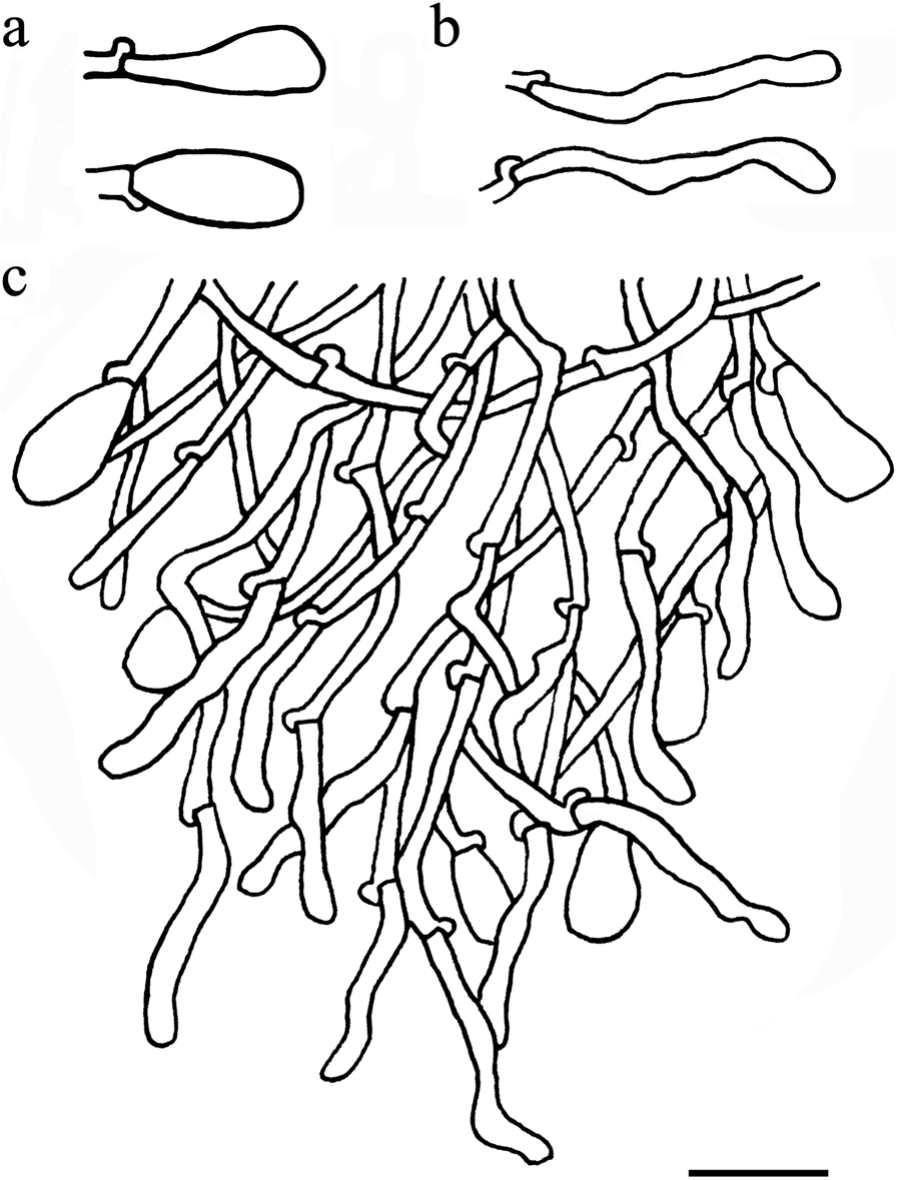
Microscopic structures of *Skvortzovia qilianensis* (drawn from holotype). **a** basidioles. **b** leptocystidia. **c** apical part of a tooth. Scale bar = 10 μm

***Skvortzovia qilianensis*** Jia Yu, Xue W. Wang, S.L. Liu & L.W. Zhou, **sp. nov.** (Figs. 10–11)

MycoBank: MB 840230.

*Etymology: qilianensis* (Lat.), refers to the Qilian Mountains.

*Diagnosis*: Characterized in the genus by the woody hard, buff-yellow to lemon-yellow and not cracked basidiomes, and odontioid hymenophores with relatively long aculei.

*Type:* **China:** *Gansu*: Zhangye: Qilian Mountains National Park: Sidalong Forest Farm, on fallen branch of *Picea*, 4 Sep. 2018, *Li-Wei Zhou*, *LWZ 20180904-16* (HMAS – holotype).

*Description: Basidiomes* annual, closely adnate, widely effused, not easily separable, thin, woody hard, cream to pale yellow when fresh, buff-yellow to lemon-yellow with age, not cracked. *Hymenophore* odontoid, composed of small, cylindrical aculei, aculei rather distant, 2–4 aculei per mm, 400–500 μm long, the higher ones slightly attenuate with a short fimbriate sterile apex. *Subiculum* not stratified, buff to honey-yellow, 100–200 μm thick. *Margin* gradually thinning out, white, abrupt with short aculei.

*Hyphal system* monomitic, generative hyphae with clamp connections. *Subiculum* composed of indistinct generative hyphae; subicular hyphae hyaline, thin-walled, frequently branched, interwoven, 2–3 μm diam. *Subhymenial hyphae* hyaline, thin-walled, frequently branched, 1.5–2.5 μm diam. *Hymenial leptocystidia* tubular with obtuse apex, numerous, hyaline, thin-walled, with a basal clamp connection, 10–30 × 1.5–2.5 μm. *Basidia* not found; basidioles rare, clavate to cylindrical, 10–14 × 4.5–5.5 μm, tapering to 2–3 um diam with a clamp connection at base. *Basidiospores* not found.

*Additional specimens examined:* **China:** *Gansu*: Zhangye: Qilian Mountains National Park: Sidalong Forest Farm, on fallen branch of *Picea*, 4 Sep. 2018, *Li-Wei Zhou*, *LWZ 20180904-20* (HMAS), *LWZ 20180904-20* (HMAS).

*Notes:* Basidiospores were not found in any of the three specimens of *S. qilianensis* studied. Traditionally, it is not a common practice to describe new species of wood-inhabiting fungi lacking basidiospores. However, if other morphological characters are taxonomically distinct, sometimes mycologists have ignored basidiospores and described the specimens as new species (Tchoumi et al. 2020).

***Skvortzoviella*** Jia Yu, Xue W. Wang, S.L. Liu & L.W. Zhou, **gen. nov.**

MycoBank: MB 840231.

*Etymology: Skvortzoviella* (Lat.), refers to the similarity to *Skvortzovia*.

*Diagnosis:* Unique in *Hymenochaetales* being characterized by a combination of resupinate and cracked basidiomes, the smooth and light-colored hymenophore, a monomitic hyphal system, tubular leptocystidia with obtuse apex, and ellipsoid basidiospores.

*Type: Skvortzoviella lenis* Jia Yu et al. 2021.

*Description: Basidiomes* annual, closely adnate, widely effused, not easily separable, thin, membranous, rarely soft, usually with a few broad cracks or cracked extensively. *Hymenophore* smooth or irregular, cream to pale yellow. *Margin* gradually thinning out, white, filamentose.

*Hyphal system* monomitic, generative hyphae with clamp connections, hyaline, thin-walled, frequently branched. *Hymenial leptocystidia* tubular with obtuse apex, hyaline, thin-walled. *Basidia* cylindrical, often with a median constriction, four sterigmata. *Basidiospores* ellipsoid, hyaline, smooth, thin-walled, acyanophilous, non-amyloid, non-dextrinoid.

*Notes:* Morphologically, *Skvortzoviella* is closely related to *Skvortzovia*; however, *Skvortzovia* also accommodates species with grandinioid to odontioid hymenophores in addition to those with smooth hymenophores (Eriksson et al. 1981; Gruhn and Hallenberg 2018).

**Fig. 12:**
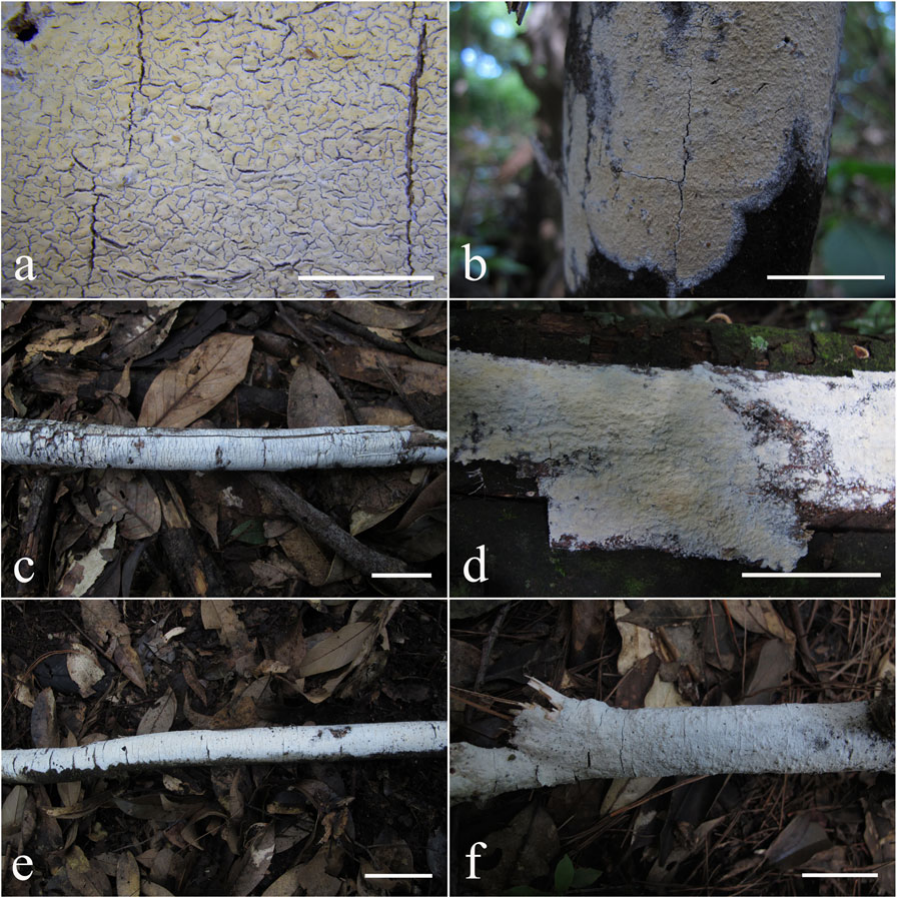
Basidiomes of *Skvortzoviella lenis*. **a**, **b** LWZ 20180921-17 (holotype). **c** LWZ 20180921-32 (paratype). **d** LWZ 20180921-25 (paratype). **e** LWZ 20180922-39 (paratype). **f** LWZ 20180922-61 (paratype). Scale bars: **a** = 5 mm; **b** = 5 cm; **c**–**f** = 3 cm

**Fig. 13:**
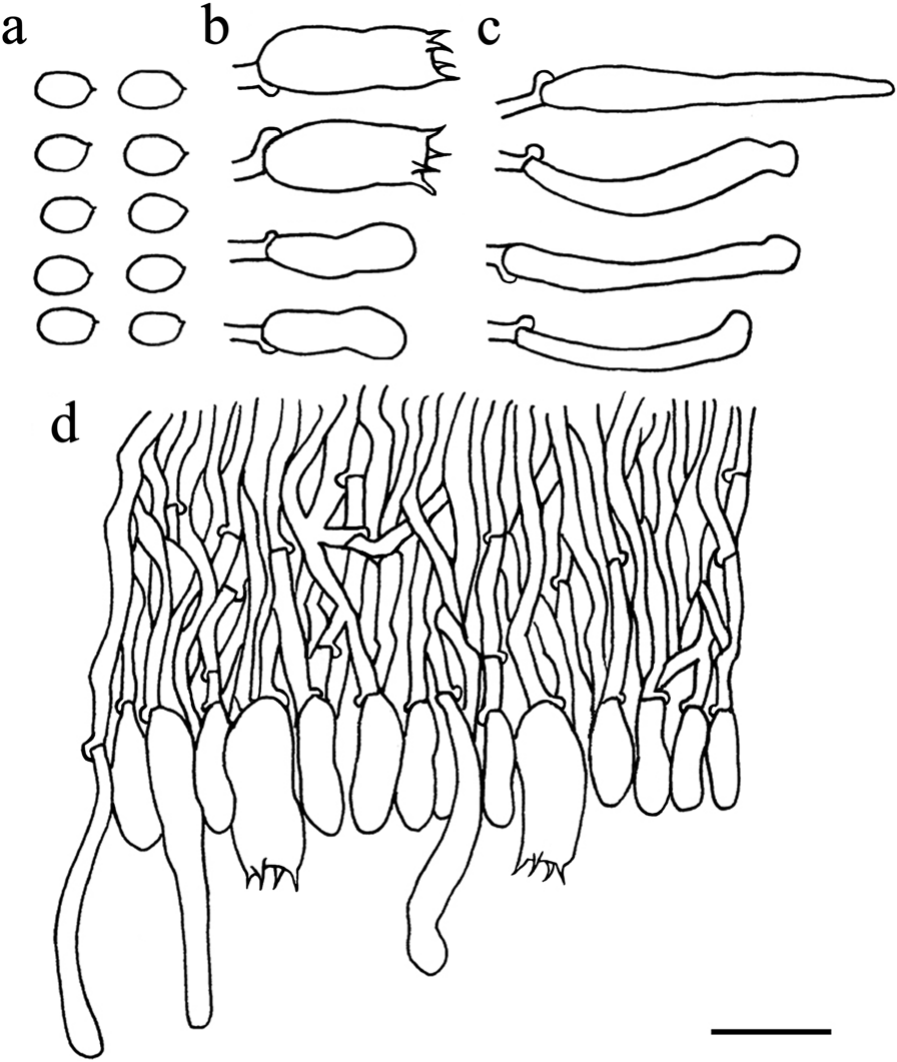
Microscopic structures of *Skvortzoviella lenis* (drawn from holotype). **a** basidiospores. **b** basidia and basidioles. **c** leptocystidia. **d** a section of hymenial and subhymenial layer. Scale bar = 10 μm

***Skvortzoviella lenis*** Jia Yu, Xue W. Wang, S.L. Liu & L.W. Zhou, **sp. nov**. (Figs. 12–13)

MycoBank: MB 840232.

*Etymology: lenis* (Lat.), refers to the smooth hymenium.

*Diagnosis*: Similar to *Skvortzovia georgica* in the smooth hymenium, but *S. georgica* has slightly larger leptocystidia (30–32 × 4–5 μm) and allantoid basidiospores (Gruhn and Hallenberg 2018).

*Type:* **China:** *Yunnan*: Baoshan: Gaoligong Mountains National Nature Reserve: Baihua Ridge, on angiosperm stump, 21 Sep. 2018, *Li-Wei Zhou*, *LWZ 20180921-17* (HMAS – holotype).

*Description: Basidiomes* annual, closely adnate, widely effused, not easily separable, thin, membranous, rarely soft, usually with a few broad cracks or cracked extensively, 50–100 μm thick. *Hymenophore* smooth or irregular, white to cream when fresh, pale yellow to buff-yellow with age. *Subiculum* not stratified, white when fresh, light cream-coloured in dried material. *Margin* gradually thinning out, white, filamentose.

*Hyphal system* monomitic, generative hyphae with clamp connections. Subiculum composed of indistinct generative hyphae; subicular hyphae hyaline, thin-walled, frequently branched, 2–2.5 μm diam. *Subhymenial hyphae* hyaline, frequently branched, compact and agglutinated, 1.5–2.0 μm diam. *Hymenial leptocystidia* tubular with obtuse apices, hyaline, thin-walled, with a basal clamp connection, 15–30 × 1.5–3 μm. *Basidia* cylindrical, often with a median constriction, four sterigmata, 12–20 × 4.7–6 μm, tapering to 2.5–3.5 μm diam with a clamp connection at base; basidioles similar in shape to basidia, but smaller. *Basidiospores* ellipsoid, hyaline, smooth, thin-walled, acyanophilous, non-amyloid, non-dextrinoid, 4.8–5.8(− 6.0) × (2.8–)2.9–3.5 μm, L = 5.1 μm, W = 3.1 μm, Q = 1.62–1.67 (*n* = 90/3).

*Additional specimens examined:* **China:** *Yunnan*: Baoshan: Gaoligong Mountains National Nature Reserve: Baihua Ridge, on fallen angiosperm twig, 21 Sep. 2018, *Li-Wei Zhou*, *LWZ 20180921-7* (HMAS); *loc. cit.,* on fallen angiosperm trunk, 21 Sep. 2018, *Li-Wei Zhou*, *LWZ 20180921-25* (HMAS); *loc. cit.,* on fallen angiosperm twig, 21 Sep. 2018, *Li-Wei Zhou*, *LWZ 20180921-32* (HMAS); *loc. cit.,* on fallen angiosperm branch, 22 Sep. 2018, *Li-Wei Zhou*, *LWZ 20180922-39* (HMAS), *LWZ 20180922-61* (HMAS).

*Notes: Skvortzovia furfuracea* resembles *Skvortzoviella lenis* in the smooth to grandinioid hymenophore and ellipsoid basidiospores, but differs by the presence of halocystidia (Eriksson et al. 1981).

## DISCUSSION

Previous phylogenies have shown the intraspecific ITS variations in *Resinicium*, such as those in *R. friabile*, *R. grandisporum,* and *R. saccharicola* (Nakasone 2007; Gruhn et al. 2017). Similarly, the newly described *R. austroasianum* formed intraspecific clades (LWZ 20180517-42 and the other four specimens; Figs. 1–2). However, these clades are short-branched and no morphological differences could be found corresponding to these clades. Therefore, we treated these genetic distances as intraspecific but not interspecific variations.

*Resinicium* was considered to originate in tropical America due to the high species diversity including basal lineages there (Nakasone 2007, Gruhn et al. 2017). However, the current study identifies two new species and more importantly a new basal lineage of *Resinicium* from tropical regions in the Asia-Pacific area (Figs. 1–2), which in part places doubt on the tropical American origin of this genus (Nakasone 2007, Gruhn et al. 2017). Moreover, the biogeographic analysis based on the combined dataset of ITS and nLSU regions also supported Asia-Pacific as the ancestral origin of *Resinicium* (Fig. 3). Comparing with the combined dataset (3), the ITS dataset of *Resinicium* (2) included more vouchers and species of *Resinicium* but failed to resolve species relationships with reliable statistical values in some lineages in biogeographic analysis (data not shown). Therefore, the current Asia-Pacific origin of *Resinicium* is not conclusive. A wider sampling around tropical regions in a multi-locus-based biogeographic analysis will clarify the geographic origin and evolution of *Resinicium*.

Like previous studies (Zhou et al. 2018; Liu et al. 2019), the current combined dataset of ITS and nLSU (1) does not resolve the relationships among families within *Hymenochaetales* (Fig. 1). Therefore, the family positions of *Resinicium*, *Skvortzovia,* and *Skvortzoviella* are still ambiguous. To solve this issue, a comprehensive phylogenetic study on the whole order with the help of multi-loci and a wider sampling should be performed, which is beyond the scope of the current study. However, the current study provides new materials for further reconstructing the phylogenetic backbone of *Hymenochaetales.*

## CONCLUSION

**Fig. 14:**
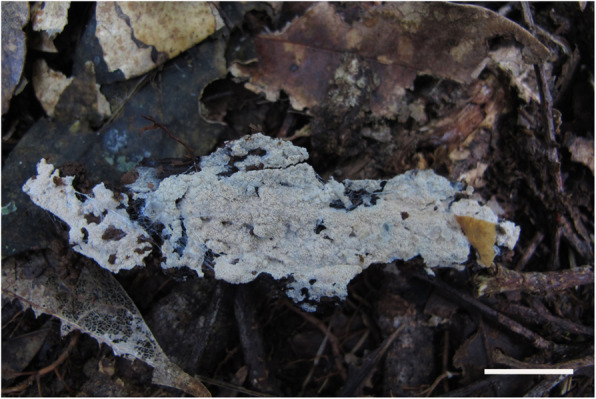
Basidiomes of *Resinicium* sp. (LWZ 20171015-31) in situ. Scale bar = 2 cm

**Fig. 15:**
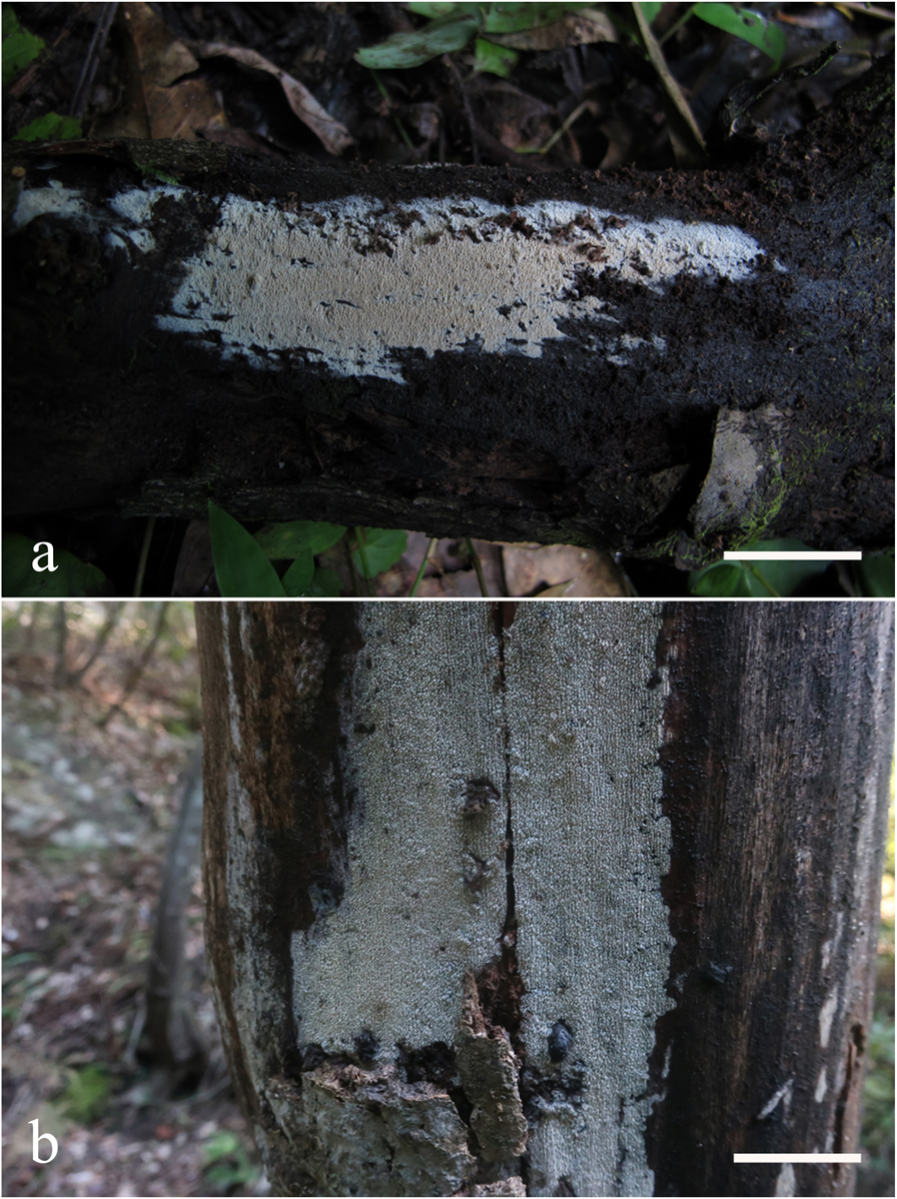
Basidiomes of *Skvortzovia pinicola* in situ. **a** LWZ 20180921-6. **b** LWZ 20201011-18. Scale bars = 2 cm

The current study revealed one new monotypic genus, *Skvortzoviella*, typified by the new species, *S. lenis*, and four other new species, viz. *Resinicium austroasianum*, *R. lateastrocystidium*, *Skvortzovia dabieshanensis* and *S. qilianensis,* from the Asia-Pacific region. Besides, a new basal lineage of *Resinicium* represented by one specimen LWZ 20171015–31 from Vietnam (Fig. 14), and three Chinese specimens of *Skvortzovia pinicola* (Fig. 15) are also identified. Phylogenetic analyses support the six new taxa and the new lineage of *Resinicium* as members of *Hymenochaetales* (Fig. 1) thereby adding to the knowledge of generic and species diversity within this order.

## Data Availability

All sequence data generated for this study can be accessed via GenBank: https://www.ncbi.nlm.nih.gov/genbank/. All alignments for phylogenetic analyses were deposited in TreeBASE (http://www.treebase.org; accession number S27463).

## ABBREVIATIONS

BI: Bayesian inference
BPP: Bayesian posterior probability
BS: Bootstrap
CB: Cotton Blue
CTAB: Cetyl-trimethyl-ammonium bromide
ESSs: Effective sample sizes
IKI: Melzer’s reagent
ITS: Nuclear ribosomal internal transcribed spacer
KOH: 5% potassium hydroxide
ML: Maximum likelihood
nLSU: Large subunit nuclear ribosomal RNA gene
PCR: Polymerase chain reaction
PSRFs: Potential scale reduction factors
